# Immediate Postmastectomy Breast Reconstruction Showed Limited Advantage in Patient Survival after Stratifying by Family Income

**DOI:** 10.1371/journal.pone.0082807

**Published:** 2013-12-12

**Authors:** Yi-Zhou Jiang, Yi-Rong Liu, Ke-Da Yu, Wen-Jia Zuo, Zhi-Ming Shao

**Affiliations:** Department of Breast Surgery, Cancer Center and Cancer Institute, Shanghai Medical College, Fudan University, Shanghai, P.R. China; The University of Hong Kong, China

## Abstract

**Background:**

Postmastectomy breast reconstruction is widely used in breast cancer patients for its aesthetic effect. Although several studies have casted suspicion upon the oncological safety of immediate breast reconstruction after mastectomy, the potential impact of different reconstruction methods on patient survival remains unclear.

**Patients and Methods:**

We identified 35,126 female patients diagnosed with breast cancer from January 1, 1998 to December 31, 2002 in the Surveillance, Epidemiology, and End Results database. Breast cancer-specific survival (BCSS) and overall survival (OS) were compared among patients who underwent mastectomy with or without immediate breast reconstruction (autologous reconstruction or implant reconstruction) using Cox proportional hazard regression models.

**Results:**

In multivariate analysis unadjusted for family income, patients undergoing immediate postmastectomy reconstruction exhibited improved BCSS [pooled reconstruction (any types of reconstruction): hazard ratio (HR)  =  0.87, 95% confidence interval (CI) 0.80–0.95, *P* = 0.001] and OS (pooled reconstruction: HR = 0.70, 95% CI 0.65–0.75, *P*<0.001) compared to patients who underwent mastectomy alone. However, after stratifying by family income, patients receiving reconstruction showed limited advantage in BCSS and OS compared with those undergoing mastectomy alone. When comparing between the two reconstruction methods, no significant differences were observed in either BCSS (implant versus autologous reconstruction: HR = 1.11, 95%CI 0.90–1.35, P = 0.330) or OS (implant versus autologous reconstruction: HR = 1.07, 95% 0.90–1.28, P = 0.424).

**Conclusions:**

Compared to mastectomy alone, immediate postmastectomy reconstruction had limited advantage in survival after adjusting for confounding factor of family income. Our findings, if validated in other large databases, may help to illustrate the actual effect of immediate postmastectomy reconstruction on patient survival.

## Introduction

Breast cancer is the most common female malignancy in both the developing and developed countries, with over 1.3 million cases diagnosed annually and almost 0.5 million deaths [1], [2]. As a major treatment protocol, mastectomy is used to treat nearly 60,000 patients diagnosed with breast cancer in the United States (US) annually [3], [4]. While this procedure may have a profound impact on the patient’s physical well-being, the surgical result of this procedure will impair a patient’s body image. Such drawbacks can be effectively remedied by breast reconstruction, especially when performed immediately after mastectomy [5]–[7]. Although several studies suggest the oncological safety of breast reconstruction by demonstrating that immediate breast reconstruction neither impedes the local recurrence [8], [9] nor delays adjuvant therapies [10], [11], the underlying interactions between grafts and residual breast tissues are ambiguous.

Recently, adipocytes have been suggested to play an important role in the origin and development of breast cancer. Yasushi Manabe and his colleagues [12] found that mature adipose cells promoted the growth of breast cancer cells in collagen gel matrix culture through their growth-promoting effect on estrogen receptor (ER)-positive tumor cells. Puneeth Iyengar’s group [13] revealed that adipocytes contributed significantly to tumor growth at early stages through secretion and processing of collagen VI. Obviously, autologous breast reconstruction would increase the number of adipocytes in the surgical region, but it remains elusive whether this lipofilling effect will impair the oncological safety of reconstruction.

In the present study, we used the US National Cancer Institute’s (NCI) Surveillance, Epidemiology, and End Results (SEER) database to obtain a population-based data. Breast cancer-specific survival (BCSS) and overall survival (OS) were comprehensively compared among patients who underwent mastectomy alone and those who underwent different methods of immediate postmastectomy reconstruction.

## Materials and Methods

### Ethics statement

Our study was approved by the independent ethical committee/institutional review board of Fudan University Shanghai Cancer Center (Shanghai Cancer Center Ethical Committee). The data released through the SEER database does not require informed patient consent because cancer is a reportable disease in every state in the US.

### Inclusion and exclusion criteria

Data were obtained from the current SEER database consisting of 18 population-based cancer registries. We selected female patients diagnosed with unilateral breast cancer from January 1, 1998, through December 31, 2002. Patients diagnosed with breast cancer before 1998 were excluded because SEER did not record reconstruction data until 1998 [14]; Patients diagnosed with breast cancer after 2002 were excluded to ensure adequate follow-up time.

We included 35,126 patients in this study according to the following criteria: female, age of diagnosis between 18 and 84 years, breast cancer as the primary and only cancer diagnosis, unilateral breast cancer, pathologically confirmed infiltrating ductal carcinoma (IDC, ICD-O-3 8500/3), AJCC stages I to III, undergoing following types of mastectomy including total mastectomy, modified radical mastectomy, radical mastectomy, extended radical mastectomy, or mastectomy otherwise unspecified (surgery of primary site code: 40–80). Patients treated with partial or subcutaneous mastectomy were excluded. Patients with histological grade IV (SEER program code: undifferentiated or anaplastic) or missing data regarding reconstruction status were also excluded.

### Data management and statistical analysis

Demographic and tumor characteristics were generated for patients who underwent mastectomy alone and those who underwent the first course of reconstruction immediately at the time of their mastectomy. The latter group of patients were further categorized into implant only and autologous only (including reconstruction with rectus abdominis flap, latissimus dorsi flap, and flap not otherwise specified) subgroups. Patients who received other types of reconstruction or received combination of autologous and implant techniques were not included in either the implant or the autologous groups.

Demographic statistics included age at diagnosis, race, marital status, family income, year of diagnosis, county metropolitan status and county education level (Table 1). Age was categorized into <45, 45–64, >64 years groups. Race and ethnicity were coded as white, black, and other (American Indian/AK Native, Asian/Pacific Islander). Marital status was coded as married and not married including divorced, widowed, single (never married) and separated. Annual family income were divided into four groups (<$4645, 4645–5116, 5117–6281, >6281) by the quartiles income of all studied cases. According to the median percent of individuals having over a 12th grade education level, county education was divided as high or low. Tumor characteristics included laterality, tumor size, histological grade, lymph nodes status, ER status, progesterone receptor (PR) status, and radiotherapy. For histological grade, grade I presented as well differentiated, grade II was moderately differentiated, and grade III was poorly differentiated.

**Table 1:** Demographic and tumor characteristics of the study sample.

	Mastectomy only	Reconstruction Type				
		All	Autologous Only	Implant Only	*P1* a	*P2* b
Variable	NO. (%)	NO. (%)	NO. (%)	NO. (%)		
	29003	6123	2649	1412		
Age, y					<0.001	0.106
<45	4460 (15.4)	2059 (33.6)	908 (34.3)	493 (34.9)		
45–64	12900 (44.5)	3559 (58.1)	1545 (58.3)	790 (55.9)		
>64	11643 (40.1)	505 (8.2)	196 (7.4)	129 (9.1)		
Race					<0.001	<0.001
White	22988 (79.6)	5273 (86.3)	2208 (83.5)	1248 (88.6)		
Black	2758 (9.5)	523 (8.6)	303 (11.5)	82 (5.8)		
Other^c^	3147 (10.9)	314 (5.1)	134 (5.1)	78 (5.5)		
Marital status					<0.001	0.904
Married	16121 (55.6)	4183 (68.3)	1815 (68.5)	961 (68.1)		
Not married^d^	11660 (40.2)	1763 (28.8)	761 (28.7)	409 (29.0)		
Unknown	1222 (4.2)	177 (2.9)	73 (2.8)	42 (3.0)		
Family income					<0.001	0.012
<$4645	6168 (21.3)	795 (13.0)	366 (13.8)	177 (12.5)		
$4645–$5116	9002 (31.0)	1394 (22.8)	592 (22.3)	362 (25.6)		
$5117–$6281	6808 (23.5)	1697 (27.7)	787 (29.7)	366 (25.9)		
>$6281	7024 (24.2)	2237 (36.5)	904 (34.1)	507 (35.9)		
Year of diagnosis					0.035	0.130
1998–2000	14996 (51.7)	3079 (50.3)	1353 (51.1)	686 (48.6)		
2001–2002	14007 (48.3)	3044 (49.7)	1296 (48.9)	726 (51.4)		
County type					<0.001	0.429
Metropolitan	24042 (82.9)	5610 (91.6)	2430 (91.7)	1285 (91.0)		
Nonmetropolitan	4961 (17.1)	513 (8.4)	219 (8.3)	127 (9.0)		
County education^e^					<0.001	<0.001
High	14203 (49.0)	3364 (54.9)	1338 (50.5)	805 (57.0)		
Low	14799 (51.0)	2759 (45.1)	1311 (49.5)	607 (43.0)		
Laterality					0.485	0.141
Right	14292 (49.3)	2995 (48.9)	1323 (49.9)	671 (47.5)		
Left	14708 (50.7)	3127 (51.1)	1326 (50.1)	741 (52.5)		
Tumor size					<0.001	<0.001
<2 cm	12742 (44.6)	3193 (52.5)	1304 (49.7)	802 (57.0)		
2–5 cm	13337 (46.7)	2502 (41.2)	1119 (42.7)	534 (38.0)		
>5 cm	2487 (8.7)	382 (6.3)	200 (7.6)	71 (5.0)		
Grade^f^					0.016	0.001
I	3611 (12.5)	732 (12.0)	283 (10.7)	196 (13.9)		
II	11274 (38.9)	2405 (39.3)	1015 (38.3)	565 (40.0)		
III	13033 (44.9)	2735 (44.7)	1248 (47.1)	596 (42.2)		
Unknown	1085 (3.7)	251 (4.1)	103 (3.9)	55 (3.9)		
Node status					<0.001	<0.001
Negative	14842 (51.2)	3323 (54.3)	1375 (51.9)	809 (57.3)		
1–3 positive	7735 (26.7)	1778 (29.0)	766 (28.9)	405 (28.7)		
>3 positive	5443 (18.8)	862 (14.1)	429 (16.2)	165 (11.7)		
Unknown	983 (3.4)	160 (2.6)	79 (3.0)	33 (2.3)		
ER^g^					0.122	0.029
Positive	17574 (60.7)	3815 (62.5)	1594 (60.4)	910 (64.6)		
Negative	6801 (23.5)	1410 (23.1)	654 (24.8)	319 (22.6)		
Unknown	4580 (15.8)	883 (14.5)	391 (14.8)	180 (12.8)		
PR^g^					0.063	0.026
Positive	14525 (50.4)	3213 (52.8)	1344 (51.0)	769 (54.9)		
Negative	9242 (32.1)	1908 (31.4)	859 (32.6)	440 (31.4)		
Unknown	5065 (17.6)	963 (15.8)	430 (16.3)	191 (13.6)		
Radiotherapy					<0.001	0.032
Yes	6393 (22.0)	1115 (18.2)	528 (19.9)	238 (16.9)		
No	21477 (74.1)	4798 (78.4)	2015 (76.1)	1125 (79.7)		
Unknown	1133 (3.9)	210 (3.4)	106 (4.0)	49 (3.5)		

^a^ P value of Chi-square test comparing the mastectomy only and the pooled reconstruction groups.

^b^ P value of Chi-square test comparing the autologous only and implant only groups.

^c^ Including American Indian/AK Native, Asian/Pacific Islander.

^d^ Including divorced, widowed, single (never married),separated.

% of individuals having over a 12th grade education level; Low indicates a county with less than 78.0% (including 78.0%) of individuals having over a 12th grade education level. (78.0% is the median of all county education data studied).^e^ High indicates a county with greater than 78.0

^f^ Grade are coded as followings: Well differentiated; Grade I; Moderately differentiated; Grade II; Poorly differentiated; Grade III; Unknown.

^g^ ER: Estrogen Receptor; PR: Progesterone Receptor.

Chi-square tests were used to evaluate the differences between mastectomy and different reconstruction types. Kaplan-Meier plots and log-rank tests were performed to compare unadjusted BCSS and OS among different treatment groups. Adjusted hazard ratios (HRs) with 95% confidence intervals (CIs) were estimated using Cox proportional hazard regression models. All the statistical analyses were performed with SPSS statistics, version 20 (SPSS, Chicago, IL, USA). A two-sided *P*<0.05 was deemed to be statistically significant.

## Results

### Demographic and tumor characteristics

A total of 35,126 patients were included in this study according to the inclusion and exclusion criteria stated above, of which 29,003 patients underwent mastectomy alone while 6,123 patients underwent immediate breast reconstruction after mastectomy. Furthermore, in the reconstruction group, 2,649 females received autologous reconstruction and 1,412 patients received implant reconstruction. The remaining 2062 females received other types of reconstruction, including reconstruction not otherwise specified (unknown if flap), abdominus recti flap plus implant, latissimus dorsi flap plus implant and flap not otherwise specified plus implant, were unsuitable to be classified into either the implant or the autologous groups. All demographic and tumor characteristics are shown in Table 1.

Patients with younger age (percentage of patients who underwent mastectomy alone versus pooled reconstruction: 15.4% vs. 33.6% for <45y, *P*<0.001), white race (79.6% vs. 86.3%, *P*<0.001), higher family income (47.7% vs. 64.2%, for >$5117, *P*<0.001), higher education level (49.0% vs. 54.9%, *P*<0.001) were more likely to undergo reconstruction. Also, women who were married at diagnosis (55.6% vs. 68.3%, *P*<0.001) and lived in metropolis (82.9% vs. 91.6%, *P*<0.001) were more likely to receive immediate reconstruction. As to tumor characteristics, the reconstruction group was associated with smaller tumor size (44.6% vs. 52.5% for <2 cm, *P*<0.001), fewer positive lymph nodes (51.2% vs. 54.3% for node negative, *P*<0.001; 26.7% vs. 29.0% for 1–3 positive nodes, *P*<0.001) and less application of radiotherapy (22.0% vs. 18.2%, *P*<0.001). Laterality (*P* = 0.485) was well balanced between the mastectomy and reconstruction groups. All demographic and tumor characteristics showed similarity between different reconstruction types (autologous only and implant only, Table 1) except race, tumor size, country education level, tumor grade and node status.

### Comparison of survival between the mastectomy group and the pooled reconstruction group

**Figure 1:**
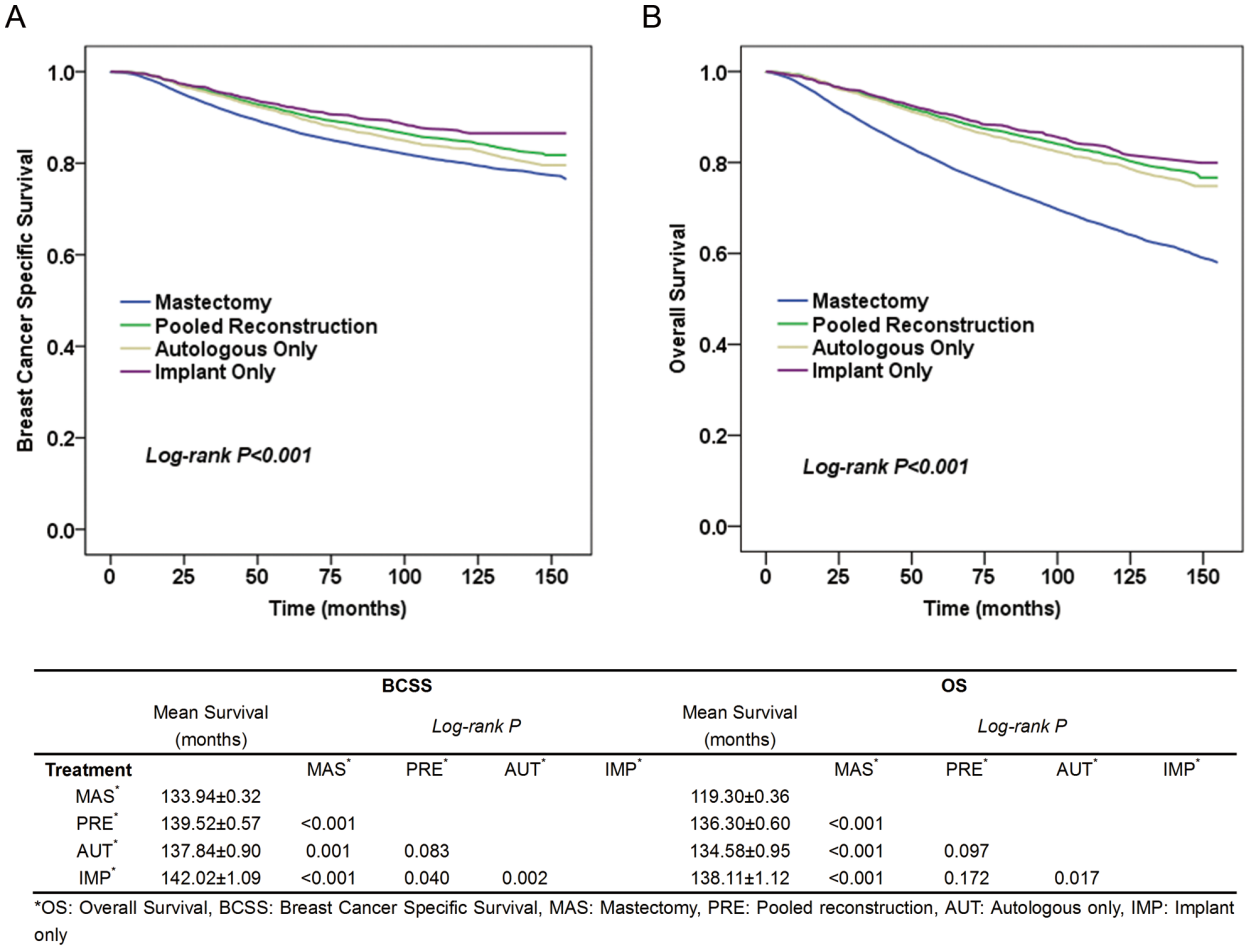
Kaplan–Meier estimates of breast cancer-specific survival and overall survival according to different treatments. **A: Breast cancer specific survival, B: Overall survival.** The table below lists the results of pairwise comparisons of breast cancer-specific survival and overall survival between different treatment groups.

We analyzed the unadjusted BCSS and OS via Kaplan-Meier plots. The median follow-up time was 107 months. Compared with patients undergoing mastectomy alone, women receiving immediate breast reconstruction had better BCSS (log-rank *P*<0.001, Figure 1). This advantage became more obvious in OS (log-rank *P*<0.001, Figure 1).

Results of BCSS and OS analysis with Cox proportional hazard regression model are shown in Table 2 and Table 3. In multivariate analysis, patients treated with immediate reconstruction had improved BCSS and OS (BCSS: HR = 0.87, 95% CI 0.80–0.95, *P* = 0.001; OS: HR = 0.70, 95% CI 0.65–0.75, *P*<0.001) compared to patients who underwent mastectomy alone. Other factors associated with improved BCSS and OS included diagnosis at age 45–64, white race, being married, diagnosis after the year 2000, smaller tumor size, lower histological grade, less positive lymph nodes, positive ER, positive PR. Patients who received radiotherapy showed better OS (*P*<0.001) but not BCSS (*P* = 0.073).The county education level, county type and laterality of the primary breast cancer had no effect on either BCSS (*P* = 0.579, 0.132, 0.507, respectively) or OS (*P* = 0.096, 0.205, 0.650, respectively).

**Table 2:** Cox proportional hazard regression model of Breast Cancer-Specific Survival.

	Univariate		Multivariate	
Variable^a^	HR (95%CI)	*P*	HR (95%CI)	*P*
Reconstruction type				
Mastectomy only	1.00	-	1.00	-
Pooled reconstruction	0.74 (0.68–0.80)	<0.001	0.87 (0.80–0.95)	0.001
Autologous only	0.83 (0.74–0.93)	0.001	0.90 (0.80–1.01)	0.065
Implant only	0.61 (0.51–0.72)	<0.001	0.80 (0.68–0.96)	0.014
Age, y				
<45	1.39 (1.30–1.49)	<0.001	1.16 (1.08–1.25)	<0.001
45–64	1.00	-	1.00	-
>64	1.16 (1.08–1.24)	<0.001	1.41 (1.32–1.52)	<0.001
Race				
White	1.00	-	1.00	-
Black	1.95 (1.80–2.12)	<0.001	1.39 (1.28–1.51)	<0.001
Other^b^	0.85 (0.77–0.95)	0.003	0.88 (0.79–0.97)	0.014
Marital status				
Married	1.00	-	1.00	-
Not married^c^	1.31 (1.24–1.39)	<0.001	1.17 (1.10–1.24)	<0.001
Year of diagnosis				
1998–2000	1.00	-	1.00	-
2001–2002	0.95 (0.90–1.01)	0.106	0.92 (0.87–0.97)	0.003
County type				
Metropolitan	1.00	-	1.00	-
Nonmetropolitan	0.98 (0.90–1.06)	0.630	1.07 (0.98–1.16)	0.132
County education^d^				
High	1.00	-	1.00	-
Low	1.18 (1.12–1.25)	<0.001	1.02 (0.96–1.08)	0.579
Laterality				
Right	1.00	-	1.00	-
Left	1.02 (0.97–1.08)	0.454	1.02 (0.96–1.08)	0.507
Tumor size				
<2 cm	1.00	-	1.00	-
2–5 cm	3.25 (3.03–3.49)	<0.001	1.93 (1.79–2.08)	<0.001
>5 cm	6.49 (5.94–7.11)	<0.001	2.72 (2.47–3.00)	<0.001
Grade^e^				
I	1.00	-	1.00	-
II	3.40 (2.86–4.04)	<0.001	2.28 (1.91–2.71)	<0.001
III	7.42 (6.27–8.77)	<0.001	3.25 (2.73–3.86)	<0.001
Node status				
Negative	1.00	-	1.00	-
1–3 positive nodes	2.45 (2.28–2.64)	<0.001	2.08 (1.93–2.25)	<0.001
>3 positive nodes	5.86 (5.46–6.29)	<0.001	4.10 (3.78–4.44)	<0.001
ER^f^				
Positive	1.00	-	1.00	-
Negative	2.29 (2.16–2.42)	<0.001	1.41 (1.30–1.53)	<0.001
PR^f^				
Positive	1.000	-	1.00	-
Negative	2.12 (2.00–2.24)	<0.001	1.38 (1.28–1.49)	<0.001
Radiotherapy				
Yes	1.00	-	1.00	-
No	0.45 (0.43–0.48)	<0.001	1.06 (0.99–1.13)	0.073

Table 2.^a^ Adjusted by Cox proportional hazards models including all factors, as categorized in

^b^ Including American Indian/AK Native, Asian/Pacific Islander.

^c^ Including divorced, widowed, single (never married),separated.

% of individuals having over a 12th grade education level; Low indicates a county with less than 78.0% (including 78.0%) of individuals having over a 12th grade education level. (78.0% is the median of all county education data studied).^d^ High indicates a county with greater than 78.0

^e^ Grade are coded as followings: Well differentiated; Grade I; Moderately differentiated; Grade II; Poorly differentiated; Grade III; Unknown.

^f^ ER: Estrogen Receptor; PR: Progesterone Receptor.

**Table 3:** Cox proportional hazard regression model of Overall Survival.

	Univariate		Multivariate	
Variable^a^	HR (95%CI)	*P*	HR (95%CI)	*P*
Reconstruction type				
Mastectomy only	1.00	-	1.00	-
Pooled reconstruction	0.49 (0.45–0.52)	<0.001	0.70 (0.65–0.75)	<0.001
Autologous only	0.54 (0.49–0.60)	<0.001	0.73 (0.66–0.81)	<0.001
Implant only	0.44 (0.38–0.50)	<0.001	0.67 (0.58–0.78)	<0.001
Age, y				
<45	1.17 (1.10–1.25)	<0.001	1.07 (1.00–1.14)	0.040
45–64	1.00	-	1.00	-
>64	2.52 (2.40–2.64)	<0.001	2.57 (2.44–2.70)	<0.001
Race				
White	1.00	-	1.00	-
Black	1.64 (1.54–1.75)	<0.001	1.33 (1.24–1.43)	<0.001
Other^b^	0.74 (0.69–0.81)	<0.001	0.80 (0.74–0.87)	<0.001
Marital status				
Married	1.00	-	1.00	-
Not married^c^	1.72 (1.65–1.80)	<0.001	1.34 (1.29–1.40)	<0.001
Year of diagnosis				
1998–2000	1.00	-	1.00	-
2001–2002	0.95 (0.90–0.99)	0.012	0.94 (0.90–0.98)	0.003
County type				
Metropolitan	1.00	-	1.00	-
Nonmetropolitan	1.10 (1.04–1.17)	0.002	1.04 (0.98–1.11)	0.205
County education^d^				
High	1.00	-	1.00	-
Low	1.21 (1.16–1.26)	<0.001	1.04 (0.98–1.11)	0.096
Laterality				
Right	1.00	-	1.00	-
Left	0.99 (0.95–1.03)	0.659	0.99 (0.95–1.03)	0.650
Tumor size				
<2 cm	1.00	-	1.00	-
2–5 cm	1.98 (1.89–2.07)	<0.001	1.51 (1.44–1.60)	<0.001
>5 cm	3.15 (2.94–3.38)	<0.001	2.07 (1.91–2.23)	<0.001
Grade^e^				
I	1.00	-	1.00	-
II	1.51 (1.39–1.65)	<0.001	1.27 (1.17–1.39)	<0.001
III	2.24 (2.07–2.43)	<0.001	1.54 (1.42–1.68)	<0.001
Node status				
Negative	1.00	-	1.00	-
1–3 positive nodes	1.53 (1.45–1.61)	<0.001	1.53 (1.45–1.61)	<0.001
>3 positive nodes	2.91 (2.77–3.07)	<0.001	2.69 (2.53–2.86)	<0.001
ER^f^				
Positive	1.00	-	1.00	-
Negative	1.57 (1.50–1.64)	<0.001	1.24 (1.16–1.32)	<0.001
PR^f^				
Positive	1.00	-	1.00	-
Negative	1.57 (1.51–1.64)	<0.001	1.25 (1.18–1.33)	<0.001
Radiotherapy				
Yes	1.00	-	1.00	-
No	0.72 (0.69–0.76)	<0.001	1.18 (1.11–1.24)	<0.001

Table 3.^a^ Adjusted by Cox proportional hazards models including all factors, as categorized in

^b^ Including American Indian/AK Native, Asian/Pacific Islander.

^c^ Including divorced, widowed, single (never married),separated.

% of individuals having over a 12th grade education level; Low indicates a county with less than 78.0% (including 78.0%) of individuals having over a 12th grade education level. (78.0% is the median of all county education data studied).^d^ High indicates a county with greater than 78.0

^e^ Grade are coded as followings: Well differentiated; Grade I; Moderately differentiated; Grade II; Poorly differentiated; Grade III; Unknown.

^f^ ER: Estrogen Receptor; PR: Progesterone Receptor.

Compared to patients who underwent mastectomy alone, patients receiving implant reconstruction had better BCSS (HR = 0.80, 95% CI 0.68–0.96, *P* = 0.014) and OS (HR = 0.67, 95% CI 0.58–0.78, *P*<0.001). However, patients treated with autologous reconstruction only experienced improved OS (HR = 0.73, 95% CI 0.66–0.81, *P*<0.001) but not BCSS (HR = 0.90, 95% CI 0.80–1.01, *P* = 0.065).

### Comparison of survival stratified by family income

We hypothesize that there might be confounding factors which would affect the relationship between reconstruction and clinical outcomes. Therefore, we further performed multivariate analysis stratifying by the potential characteristics, such as age, ER status, node status and tumor size (data not shown) and found only family income to be a confounding factor. After stratifying by family income (Table 4), only patients with income more than $6,281 demonstrated slightly improved BCSS in both the pooled reconstruction group (HR = 0.85, 95% CI 0.73–0.99, *P* = 0.034) and the implant group (HR = 0.66, 95% CI 0.47–0.91, *P* = 0.010), but not in the autologous group (HR = 0.94, 95% CI 0.77–1.15, *P* = 0.553). Also patients with income between 4,645 and 5,116 in the pooled reconstruction group experienced limited advantage in BCSS (HR = 0.83, 95% CI 0.70–0.99, *P* = 0.040). No difference in survival was observed between the remaining groups. Thus, immediate postmastectomy breast reconstruction showed limited advantage in patient survival after stratifying by family income.

**Table 4:** Cox proportional hazard regression model of Breast Cancer-Specific Survival comparing reconstruction method to mastectomy alone stratified by family income.

	Pooled Reconstruction		Autologous Only		Implant Only	
Variable^a^	HR (95%CI)	*P*	HR (95%CI)	*P*	HR (95%CI)	*P*
Family income						
<$4645	0.85 (0.67–1.08)	0.178	0.72 (0.50–1.03)	0.070	0.91 (0.56–1.46)	0.684
$4645–$5116	0.83 (0.70–0.99)	0.040	0.79 (0.61–1.02)	0.067	0.85 (0.62–1.17)	0.326
$5117–$6281	0.91 (0.78–1.07)	0.243	0.95 (0.78–1.16)	0.636	0.89 (0.64–1.24)	0.497
>$6281	0.85 (0.73–0.99)	0.034	0.94 (0.77–1.15)	0.553	0.66 (0.47–0.91)	0.010

Table 2.^a^ Adjusted by Cox proportional hazards models including all factors, as categorized in

### Comparison of survival between the subgroups of reconstruction

To further explore the impact of different reconstruction methods on patient outcome, Cox proportional hazard regression models were performed with implant reconstruction group as reference (Table 5). In univariate analysis, autologous reconstruction was associated with poorer BCSS (HR = 1.36, 95% CI 1.11–1.67, *P* = 0.003) and OS (HR = 1.24, 95% CI 1.04–1.47, *P* = 0.018). However, this association did not present in multivariate analysis in either BCSS (HR = 1.11, 95% CI 0.90–1.35, *P* = 0.330) or OS (HR = 1.07, 95% CI 0.90–1.28, *P* = 0.424).

**Table 5:** Cox proportional hazard regression model comparing reconstruction method to implant only.

	Breast Cancer-Specific Survival				Overall Survival			
	Univariate		Multivariate		Univariate		Multivariate	
Variable^a^	HR (95%CI)	*P*	HR (95%CI)	*P*	HR (95%CI)	*P*	HR (95%CI)	*P*
Reconstruction type								
Pooled reconstruction	1.21 (1.01–1.46)	0.041	1.08 (0.89–1.30)	0.438	1.12 (0.95–1.31)	0.173	1.03 (0.88–1.21)	0.689
Autologous only	1.36 (1.11–1.67)	0.003	1.11 (0.90–1.35)	0.330	1.24 (1.04–1.47)	0.018	1.07 (0.90–1.28)	0.424
Implant only	1.00	-	1.00	-	1.00	-	1.00	

Table 2, and family income.^a^ Adjusted by Cox proportional hazards models including all factors, categorized in

## Discussion

By using data from the SEER database and dividing patients into subgroups according to demographic and tumor characteristics, we were able to analyze the impact of different breast reconstruction methods on survival in a wide range of patients. Our findings suggest that immediate postmastectomy breast reconstruction shows limited advantages in BCSS in breast cancer patients after stratifying by family income. Furthermore, no statistical difference in either BCSS or OS was observed between the autologous reconstruction group and the implant reconstruction group.

Several previous studies have demonstrated that immediate postmastectomy reconstruction was correlated with better survival in breast cancer patients. A study using data from the Danish Breast Cancer Cooperative Group, included 580 implant reconstructed breast cancer patients and 1,158 individually matched controls, discovered significantly improved disease-free survival (HR = 0.78, 95% CI 0.6–0.95) in reconstructed patients [15]. Bezuhly *et al*. [5] also revealed improved BCSS among breast cancer patients undergoing immediate reconstruction in their analysis of the SEER database. Jayant Agarwal and his colleagues [7] found that patients who underwent reconstruction after mastectomy had a higher BCSS than those undergoing mastectomy alone, when controlling for demographic and oncologic covariates. However, none of these studies examined the influence of patients’ socioeconomic factors on survival. In the present study, women received reconstruction showed better BCSS and OS in multivariate analysis after adjusting for demographic and clinicopathological variables. Further stratifying patients by family income, however, we observed slightly improved BCSS only in patients with higher income. Combined with previous studies, our results demonstrated that the improved survival outcomes were largely attributable to patients’ family income. A possible explanation was that with higher family income, patients were more likely to undergo reconstruction [5]–[7] (Table 1) and have access to better medical service (e.g. neoadjuvant or adjuvant chemotherapy, adjuvant hormonal therapy, molecularly targeted therapy), which had profound effects on survival [16]–[18].

Recent studies suggested that adipocytes had positive roles in the origin and development of breast cancer. Yasushi Manabe *et al.* [12] found that mature adipose cells can promote the growth of breast carcinoma cells in collagen gel matrix culture. Petit *et al.* [19] designed a matched-cohort study including 59 lipofilled patients and 118 matched controls using the European Institute of Oncology database, and a higher risk of local event was observed in patients undergoing lipofilling. In our present study, we found that autologous reconstruction was associated with decreased BCSS and OS in univariate analysis. Thus we hypothesized that the autologous reconstruction group may demonstrate worse outcome than the implant group because of the increased number of adipocytes in the surgical region. After adjusting for demographic and tumor characteristics, we failed to observe any significant differences in either BCSS or OS between the autologous group and the implant group. A reasonable explanation is that additional adipocytes brought to the site by autologous reconstruction could promote local recurrence, but this does not significantly impair patient survival. However, we could not examine local recurrence in different reconstruction groups since the SEER database lacked this information, and a longer follow-up period would be required to demonstrate the difference in rate of local recurrence amongst the two reconstruction methods.

Compared with the prior SEER based studies [5]–[7], our study differs in several critical aspects. First, our study has an adequate follow-up time with median follow-up time of 107 months, ensuring more reliable results. Second, we adjusted the impact of socioeconomic factors (including county type, county education level) on survival and stratified patients by annual family income, revealing that family income was an important confounder for survival outcome. Furthermore, we compared survival between different reconstruction methods, revealing that there was no statistical difference in survival amongst the two methods.

Inevitably, our study has several limitations. First, the SEER database does not include data on comorbidities (e.g. cardiac and pulmonary disease). Such comorbidities can impact patient outcomes and may distribute unequally in different age groups, being less common in the younger population. We performed Cox regression analysis stratified by age, trying to minimize the impact of these factors, but they would still affect the accuracy of our analysis. Second, several important tumor characteristics (e.g. human epidermal growth factor receptor-2), the application of therapy (neoadjuvant and adjuvant), and patient outcome (recurrence and metastasis) variables are unrecorded in the SEER database, thus we could not adjust these potential confounding factors. Particularly, we could not examine the impact of different reconstruction methods on the local recurrence of breast cancer. Furthermore, since SEER only provided immediate reconstruction data, patients who received delayed reconstruction were not included in this study. Finally, our study was performed using retrospective database rather than prospective cohorts; this approach might introduce unaccounted biases.

In conclusion, our findings reveal that immediate postmastectomy reconstruction has limited advantage in patient survival after stratifying by the factor of family income. Autologous reconstruction does not impair the survival outcome. Further pre-clinical and clinical study should attempt to confirm these conclusions and clarify the underlying mechanism of the interaction between reconstruction, especially autologous reconstruction, and survival.
